# P-1599. Evaluating the Impact of Pneumococcal Urinary Antigen on Antibiotic Prescribing in an Urban Safety Net Health System

**DOI:** 10.1093/ofid/ofae631.1766

**Published:** 2025-01-29

**Authors:** Mehak Bhatia, Paul Kim, Jyothik Inampudi, Mazhar Shapoo, Marco R Scipione, Sorabh Dhar, Lea Monday

**Affiliations:** Wayne State UNiversity, Southgate, MI, Michigan; DMC/Wayne State University, Detroit, Michigan; Detroit Medical Center, Detroit, Michigan; Detroit Medical Center, Detroit, Michigan; Detroit Receiving Hospital, Detroit, Michigan; Wayne State University/Detroit Medical Center, John Dingell VAMC, Detroit, Michigan; Wayne State University School of medicine, Birmingham, Michigan

## Abstract

**Background:**

Pneumococcal urinary antigen (UAg) testing is for certain high-risk community acquired pneumonia (CAP) patients. Prior studies evaluating antibiotic de-escalation practices in patients with positive UAg are mixed^1,2^.

**Figure:**
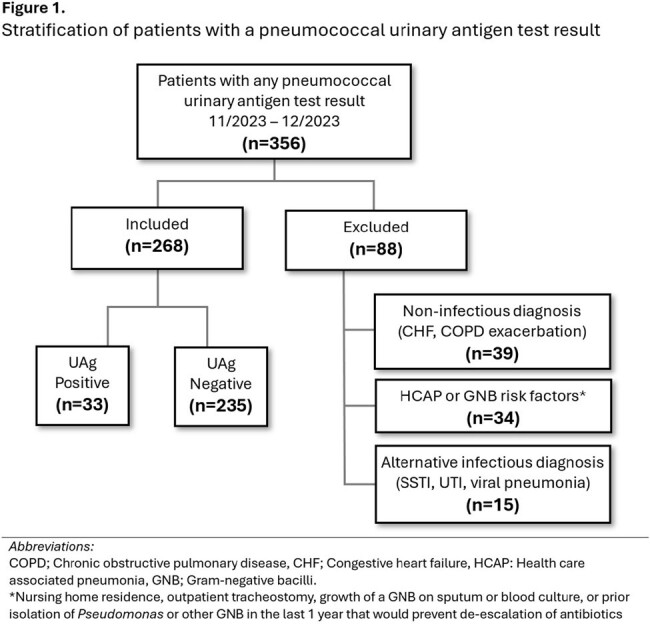

**Methods:**

We performed a retrospective cohort study of adult patients admitted with CAP who had a UAg test result over a 2-month period. Patients with an alternative diagnosis or with risk factors for healthcare associated infections that would impact de-escalation were excluded (Fig1). Patients with and without a positive UAg test result were compared. Rates of empiric versus final antibiotic prescribing and duration of empiric coverage for *Pseudomonas* and Methicillin-resistant *Staphylococcus aureus* (MRSA), were recorded. Primary outcome was whether UAg positivity impacted antibiotic de-escalation practices.

**Figure:**
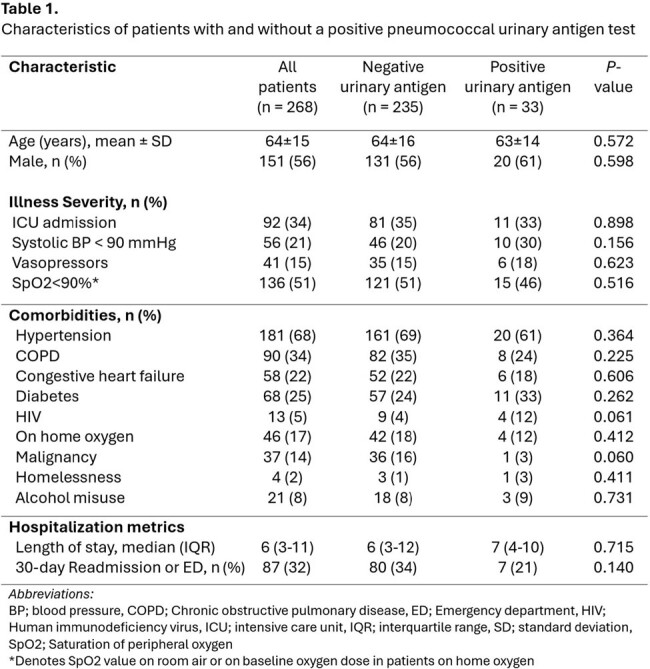

**Results:**

268 CAP patients were included in the analysis (235 UAg negative and 33 UAg positive). Cohorts were evenly matched in terms of illness severity, comorbidities, and readmission rates (Table 1). Empiric coverage for Pseudomonas and MRSA was above 40% in both cohorts. Patients started on empiric azithromycin, doxycycline, vancomycin, cefepime, or any anti-pseudomonal ß-lactam were often de-escalated, regardless of whether UAg was positive or negative (*P*< 0.05 in both cohorts) (Fig2). Empiric quinolones were more often de-escalated in UAg positive patients, though sample size was small (*P*=0.021). Definitive use of a narrow spectrum penicillin (PCN) or ß-lactam was low, even in UAg positive patients (Fig2). Duration of empiric anti-pseudomonal use was lower in UAg positive versus negative patients (median 2.5 versus 5 days, respectively)( *P*=0.0391). Shorter empiric Anti-MRSA durations were also seen in UAg positive patients (median 1 versus 4 days, respectively) (*P*=0.0201).

**Figure:**
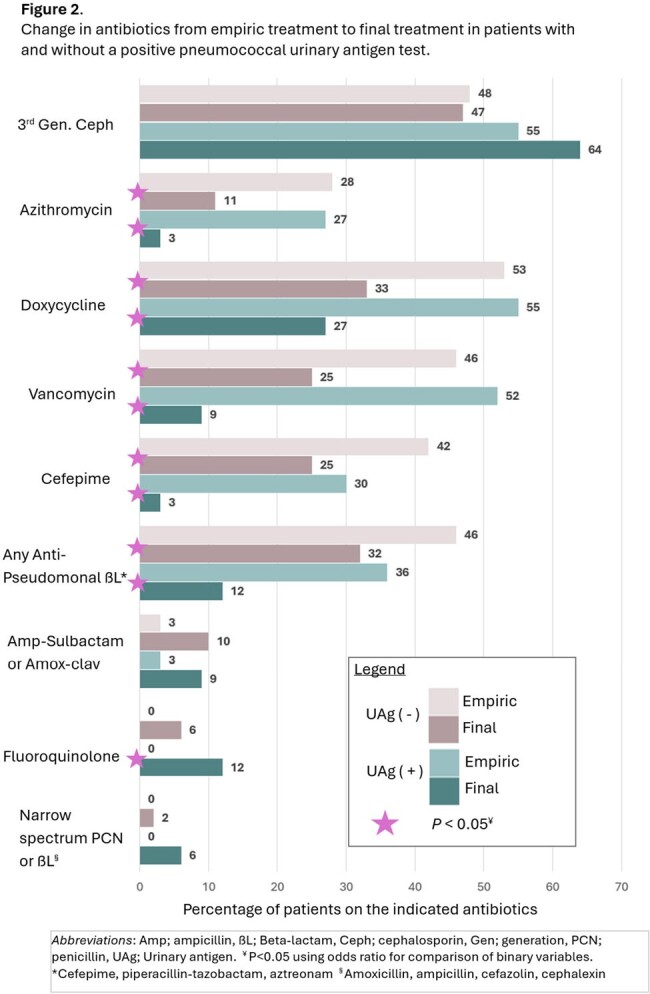

**Conclusion:**

Patients treated for CAP in an urban safety net hospital often had their empiric antibiotics de-escalated regardless of pneumococcal UAg result. However, positive UAg likely facilitated sooner discontinuation of anti-MRSA and anti-Pseudomonal coverage since median durations were lower in this group. Efforts to increase use of a narrow spectrum PCN or ß-lactam in such patients are an opportunity for future stewardship interventions.

**Figure:**
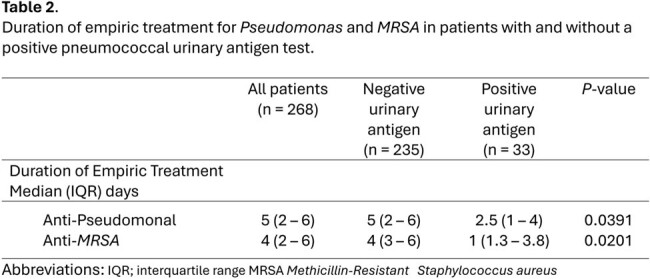

**Disclosures:**

**All Authors**: No reported disclosures

